# Supplementary material for: Genetic dissection of rapid proteolysis identifies TXNDC15 as a key factor of ERAD and lipid homeostasis
Source: bioRxiv. 2026 Apr 2:2026.04.01.715723. Preprint. [Version 1] doi: 10.64898/2026.04.01.715723 (PMC13060290; doi:10.64898/2026.04.01.715723)
Supplement: Supplement 2 [file NIHPP2026.04.01.715723v1-supplement-2.pdf]

bioRxiv preprint doi: https://doi.org/10.64898/2026.04.01.715723; this version posted April 2, 2026. The copyright holder for this preprint (which was not certified by peer review) is the author/funder, who has granted bioRxiv a license to display the preprint in perpetuity. It is made available under aCC-BY 4.0 International license.

Supplementary materials

Fig. S1

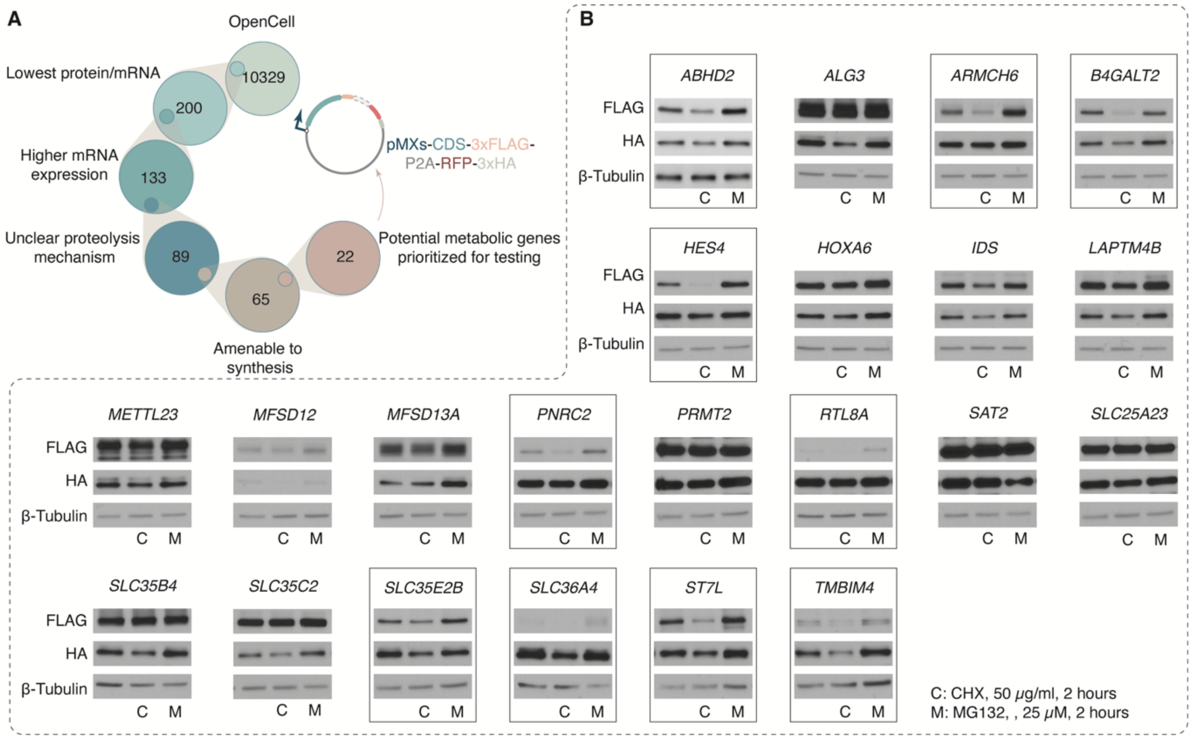

Fig. S1. Schematic for the selection and validation of short-half-life protein candidates.

(**A**) Diagram illustrating the selection and prioritization of short-half-life protein candidates from the *OpenCell* database, and the schematic of the reporter plasmid co-expressing the 3xFLAG-tagged coding sequence (CDS) of the candidate protein and 3xHA-tagged RFP from the same ORF, separated by a self-cleaving P2A peptide. (**B**) Immunoblots showing the indicated proteins from HEK293T cells transfected with reporter plasmids expressing the indicated short half-life candidate proteins. Cells were treated with cycloheximide (50 µg/ml), MG-132 (25 µM) or DMSO as a control for 2 hours prior to harvesting. β-tubulin was used as a loading control. Proteins showing behaviors consistent with proteasome-dependent degradation are highlighted by boxes.

bioRxiv preprint doi: https://doi.org/10.64898/2026.04.01.715723; this version posted April 2, 2026. The copyright holder for this preprint (which was not certified by peer review) is the author/funder, who has granted bioRxiv a license to display the preprint in perpetuity. It is made available under aCC-BY 4.0 International license.

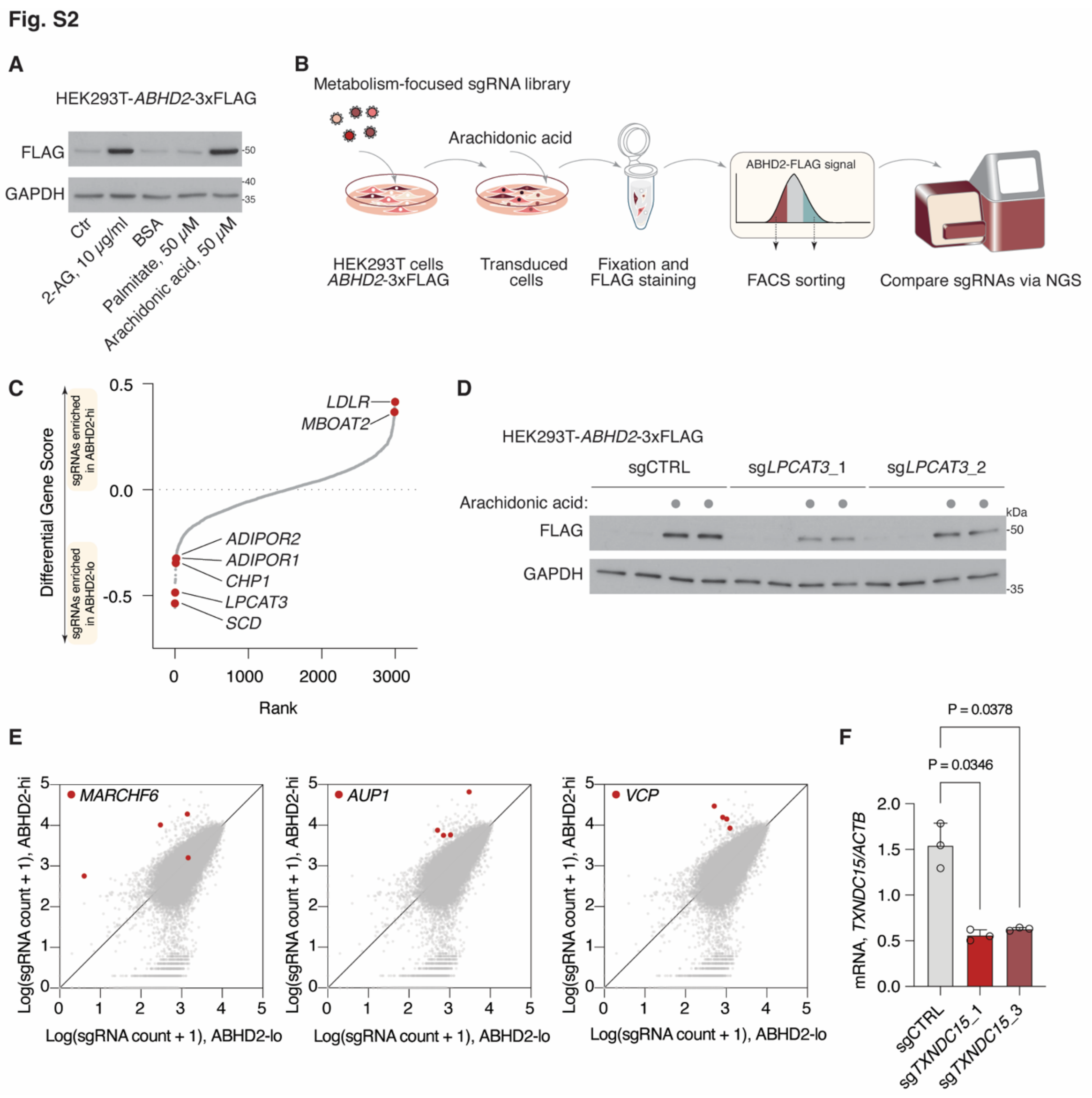

Fig. S2. ABHD2 is degraded via the MARCHF6-dependent ERAD pathway in a lipid-sensitive manner.

(**A**) Immunoblots showing the indicated proteins from HEK293T cells stably expressing ABHD2-3xFLAG, treated with the indicated lipid species for 4 hours or bovine serum albumin (BSA) as a control. β-tubulin was used as a loading control. (**B**) Schematic showing the workflow of the metabolism gene-focused, FACS-coupled CRISPR screen for regulators of ABHD2-3xFLAG stability. (**C**) Dot plot representing the CRISPR screen result. Points indicate the median fold change of sgRNA counts (gene score) of the quantified genes in the ABHD2-high versus the ABHD2-low fraction. (**D**) Immunoblots showing the indicated proteins from HEK293T cells stably expressing ABHD2-3xFLAG and transfected with plasmids expressing the sgRNAs targeting *LPCAT3* or intergenic control, treated with arachidonic acid or bovine serum albumin (BSA) as a control. GAPDH was used as a loading control. (**E**) Scatter plots showing the abundance of sgRNAs from the genome-wide CRISPR screen in the ABHD2-high versus the ABHD2-low fractions. sgRNAs targeting the indicated genes were highlighted in red. (**F**) Relative amount of *TXNDC15* mRNA in HEK293T cells transfected with plasmids expressing the indicated sgRNAs, as normalized by the mRNA levels of *ACTB*.

bioRxiv preprint doi: https://doi.org/10.64898/2026.04.01.715723; this version posted April 2, 2026. The copyright holder for this preprint (which was not certified by peer review) is the author/funder, who has granted bioRxiv a license to display the preprint in perpetuity. It is made available under aCC-BY 4.0 International license.

Fig. S3

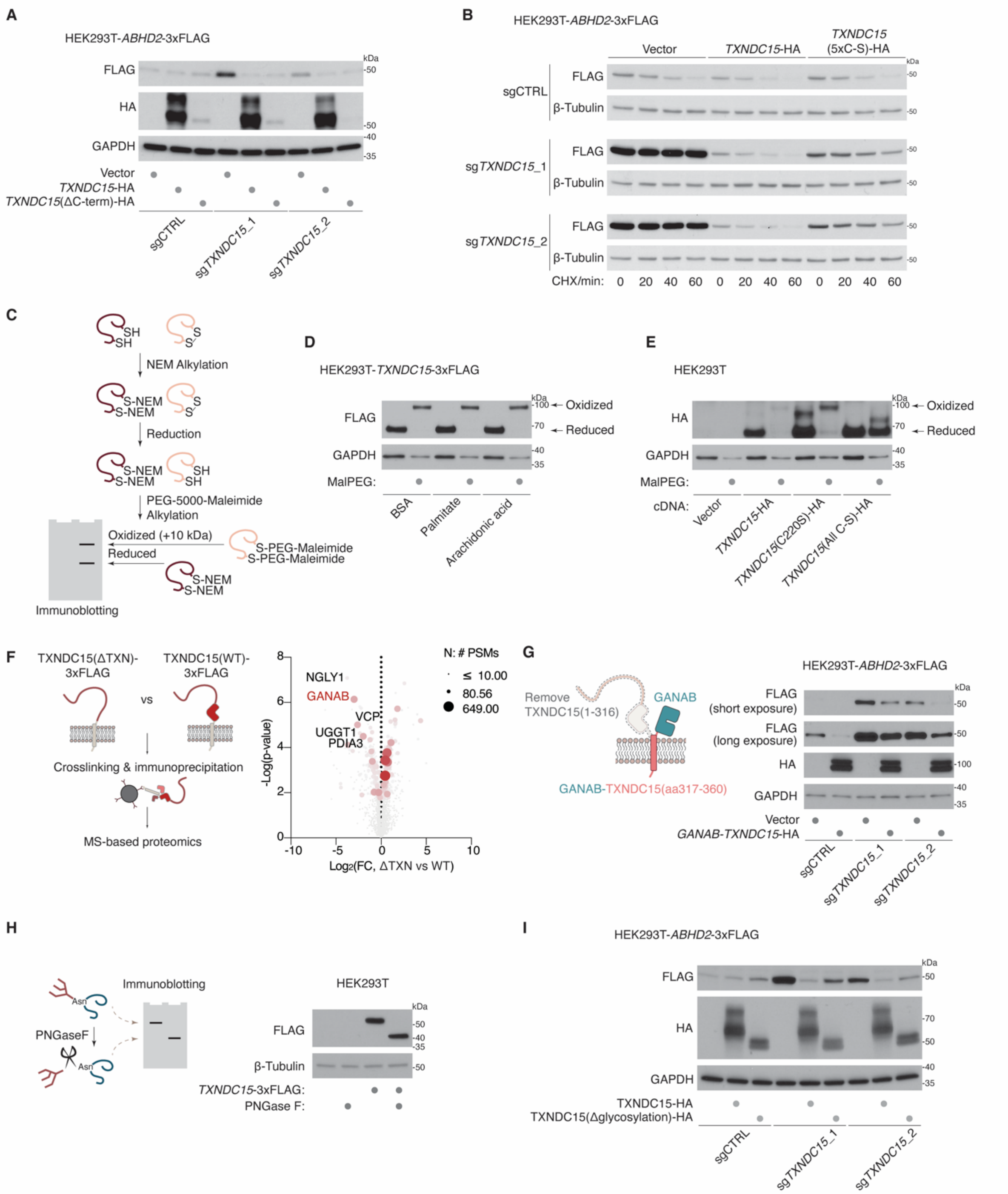

bioRxiv preprint doi: https://doi.org/10.64898/2026.04.01.715723; this version posted April 2, 2026. The copyright holder for this preprint (which was not certified by peer review) is the author/funder, who has granted bioRxiv a license to display the preprint in perpetuity. It is made available under aCC-BY 4.0 International license.

Fig. S3. TXNDC15 promotes MARCHF6 substrates degradation via catalysis-independent mechanisms.

(**A**) Immunoblots of the indicated proteins from HEK293T cells stably expressing *ABHD2*-3xFLAG cDNA and the indicated sgRNAs. Cells were complemented with cDNA of wild-type or the C-terminal truncated (Δaa350-360) mutant *TXNDC15*-HA. GAPDH was used as a loading control. (**B**) Degradation kinetics of ABHD2-3xFLAG as shown by immunoblots of the indicated proteins from HEK293T cells stably expressing *ABHD2*-3xFLAG cDNA, treated with cycloheximide (CHX, 50 µg/ml) or DMSO as a control for the indicated time. Cells were stably expressing the indicated sgRNAs and *TXNDC15*-HA, *TXNDC15*(5xC-S)-HA cDNA or an empty vector as a control. GAPDH was used as a loading control. (**C**) Schematic for the Maleimide-PEG5000 labeling assay for detecting cysteine oxidation. (**D**) Immunoblots of the indicated proteins from HEK293T cells stably expressing *TXNDC15*-3xFLAG cDNA, labeled with Maleimide-PEG5000 or N-ethylmaleimide as a control. Cells were treated with 50 µM of the indicated lipids for 8 hours. (**E**) Immunoblots of the indicated proteins from HEK293T cells stably expressing an empty vector or the indicated variants of *TXNDC15*-3xFLAG cDNA, labeled with Maleimide-PEG5000 or N-ethylmaleimide as a control. (**F**) Left, schematic of the TXNDC15 constructs used for immunoprecipitation-mass spectrometry-based proteomics analysis. Right, volcano plots showing the fold change and -Log10 p-value of protein abundance that co-immunoprecipitated with wild-type or thioredoxin-null TXNDC15 constructs. Bubble size and color indicate the summed peptide spectrum counts (Σ PSM) of the indicated protein. (**G**) Left, schematic of the wild-type TXNDC15 and GANAB-TXNDC15 chimeric constructs used in the experiment. Right, immunoblots of the indicated proteins from HEK293T cells stably expressing *ABHD2*-3xFLAG cDNA and the indicated sgRNAs. Cells were complemented with cDNA of wild-type *TXNDC15*-HA or chimeric *GANAB-TXNDC15*-HA. GAPDH was used as a loading control. (**H**) Left, schematic of the PNGaseF assay for detecting protein N-glycosylation. Right, Immunoblots of the indicated proteins from HEK293T expressing *TXNDC15*-3xFLAG cDNA or an empty vector as control. Protein lysate was treated with PNGaseF or mock-treated as a control. (**I**) Immunoblots of the indicated proteins from HEK293T cells stably expressing *ABHD2*-3xFLAG cDNA and the indicated sgRNAs. Cells were complemented with cDNA of wild-type or the glycosylation-defective (N194Q) mutant *TXNDC15*-HA. GAPDH was used as a loading control.

bioRxiv preprint doi: https://doi.org/10.64898/2026.04.01.715723; this version posted April 2, 2026. The copyright holder for this preprint (which was not certified by peer review) is the author/funder, who has granted bioRxiv a license to display the preprint in perpetuity. It is made available under aCC-BY 4.0 International license.

Data S1. Selection of candidate short-half-life genes

Data S2. Results of all CRISPR screens

Data S3. IP-MS proteomics of TXNDC15 interaction partners, TXNDC15-WT vs TXNDC15(deltaTXN)Data S2. Results of all CRISPR screens

Data S4. ER-IP proteomics, sg*TXNDC15* or sg*MARCHF6* vs sgCTRL

Data S5 Lipidomics analysis for HEK293T cells, sgCTRL vs sg*TXNDC15*
